# Early Reduction of an Open Extruded Talus: Case Report

**DOI:** 10.1055/s-0042-1744489

**Published:** 2022-04-25

**Authors:** Carlos A. Sánchez, Daniela Gutierrez, Whendy A. Mendoza, Manuel E. Niño

**Affiliations:** 1 Hospital Universitario San Ignacio, Pontificia Universidad Javeriana, Bogotá, Colômbia; 2 Faculdade de Medicina, Pontificia Universidad Javeriana, Bogotá, Colômbia; 3 Universidad de la Sabana, Cundinamarca, Colômbia; 4 Cirurgia de Pé e Tornozelo, Hospital Universitario de la Samaritana, Pontificia Universidad Javeriana, Bogotá, Colômbia

**Keywords:** avascular necrosis, fractures, bone, infections, talus

## Abstract

Talar dislocation is an infrequent lesion, with variable outcomes reported in case reports and case series. Its epidemiology has not been elucidated to date, as this lesion is described in different ways: complete talar extrusion, closed or open dislocation, open dislocation with associated talar fracture, or open dislocation with malleolar fracture. Such classifications limit the possibility of evaluating this condition as a single pathology. There is also no consensus on which is the best treatment for this lesion. Many different treatment techniques have been described, including reimplantation with and without external fixation, early osteosynthesis, and even early talectomy and tibiocalcaneal pseudoartrhodesis. The outcomes of this type of injury can be as varied as the treatment options. The complications observed in the first year after the injury can be infection, avascular necrosis (AVN) and early posttraumatic osteoarthritis. The present paper reports adequate functional and radiological outcomes after one year of early reduction of a complete talar extrusion with osteosynthesis of a medial malleolar fracture.

## Introduction

Talar extrusions result from high energy trauma, and the reports in the literature are scarce. It is estimated to comprise 0.06% of all dislocations and 2% of all talar injuries. 1 2 3 It was first described in 1680 by Fabricius Hildanus and, centuries later, in 1919, by Anderson as “aviator's astragalus” when he found this injury pattern in pilots after plane crashes. 4 Though many injury patterns are classified as “talar dislocation,” talar extrusion is defined as complete dissociation of the talus from the tibiotalar, talonavicular, and talocalcaneal joints, usually accompanied by talar or malleolar fractures with or without an associated wound. 5 6

The unique anatomical characteristics of the talus can predispose it to certain injuries and complications, such as avascular necrosis (AVN). 6 The presence of multiple articular surfaces (60% to 80% of the bone is covered by cartilage) limits the bone surface for nutritious vessels, and the absence of muscular insertions makes it prone for dislocation in high-energy trauma. 7 8 9

Although to date there is no consensus regarding the optimal treatment for isolated talar extrusion, early and delayed reimplantation with or without supplementary fixation have been reported. 1 2 3 7 8 10 11 12 For this reason, we herein present the case of an adult male with complete talar extrusion after a high-energy trauma, treated with acute reimplantation with fixation of the medial malleolar fracture.

## Case Report

The present work was approved by the Ethics in Research Committee of Hospital Universitario de la Samaritana.

**Fig. 1:**
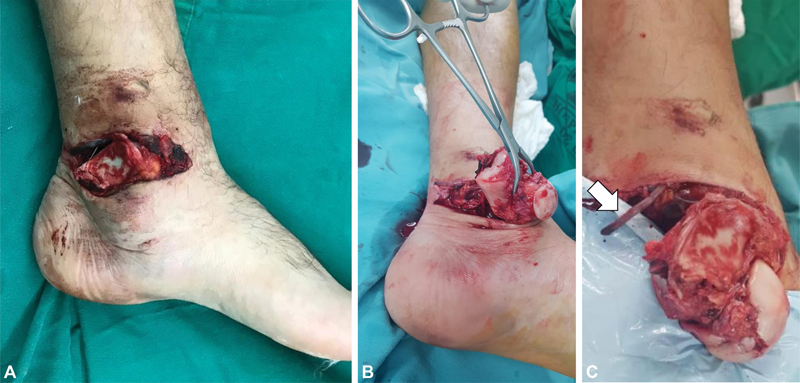
( **A** , **B** ) Complete open talar extrusion, 270° of talar rotation, no evidence of talar fracture. ( **C** ) The posterior tibial vessels are thrombosed, and not fit for repair (arrow).

**Fig. 2:**
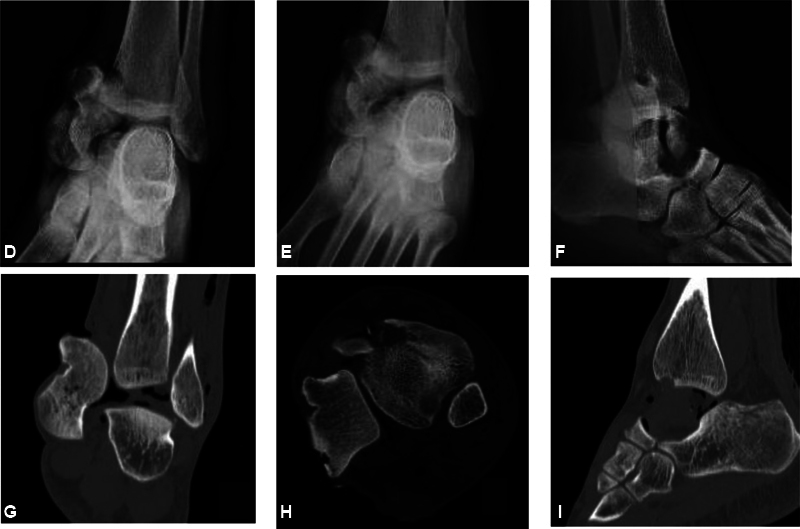
X-ray showing complete talar dislocation and tibial malleolar fracture in anteroposterior, mortise, and lateral views ( **D-F** ). Computed tomography scan detailing absence of fractures in the talus, as well as complete medial dislocation in coronal, axial, and sagittal projections ( **G-I** ).

**Fig. 3:**
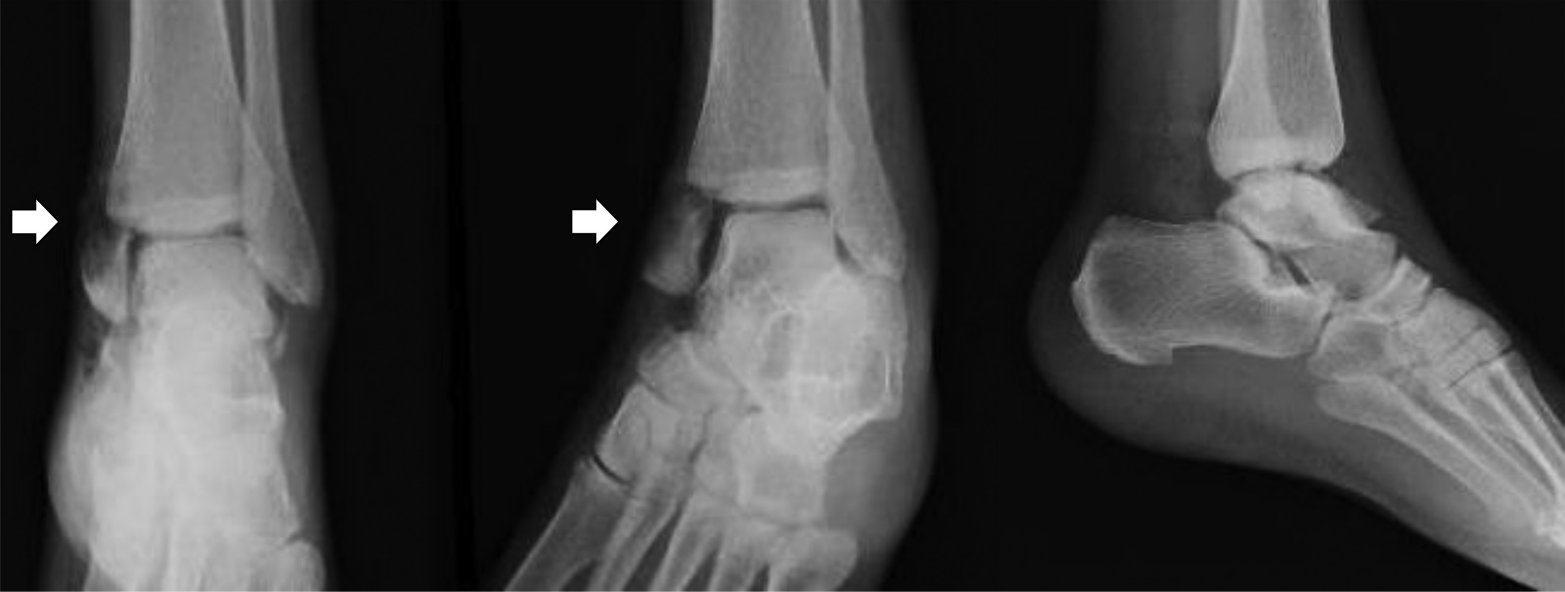
Postreduction X-ray. The talus has recovered its position, and the tibial malleolar fracture is now more evident (arrows).

**Fig. 4:**
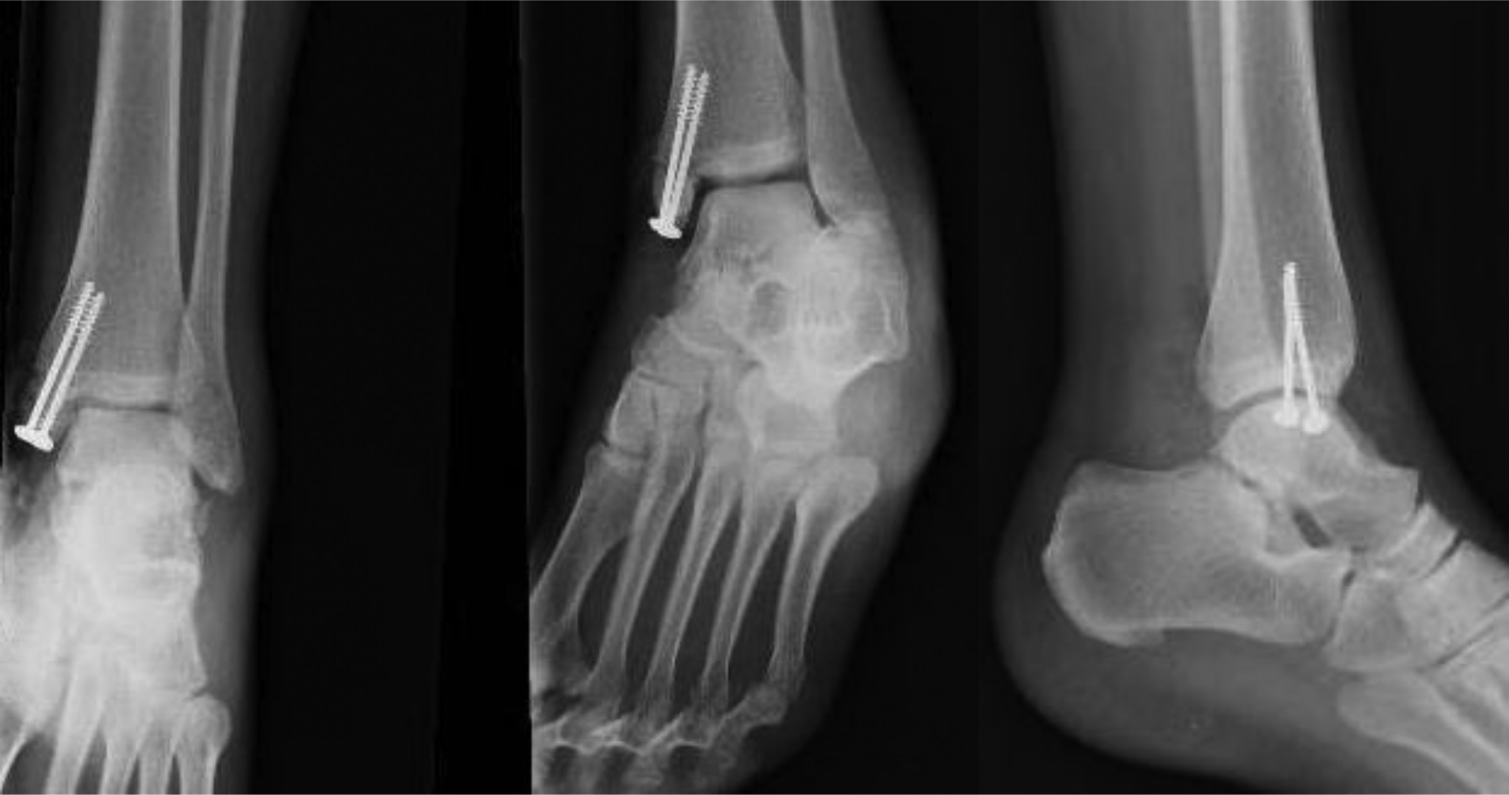
Postreduction X-ray after final fixation of the tibial malleolar fracture 48 hours after the initial reduction.

**Fig. 5:**
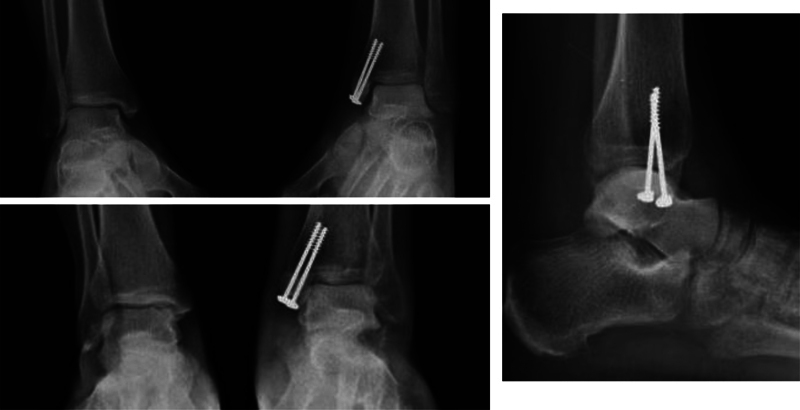
Weight-bearing X-ray after one year of follow-up. There is no evidence of avascular necrosis of the talar dome nor early signs of tibiotalar arthrosis. The tibial malleolar fracture has achieved complete consolidation.

A 26-year-old male presented with an open talar extrusion after a motorcycle accident. A 10-cm wound was evident in the medial aspect of the ankle, through which the talus was partially extruded ( Fig. 1 ). The patient was hemodynamically stable, with no other lesions observed on the initial evaluation. The foot had adequate sensibility, distal perfusion by a palpable pedal pulse, but absent posterior tibial pulse. The initial X-rays revealed a transverse tibial malleolar fracture, medial talar dislocation of 270°, and a computed tomography (CT) scan did not reveal additional fractures in the talus ( Fig. 2 ). The initial treatment consisted of intravenous antibiotics (cefazolin, gentamicin, and penicillin G), debridement, and open reduction in the operating room eight hours after the accident. Complete section of the deltoid ligament, posterior tibial artery, and vein were found during the surgical exploration. This vascular injury was not found to be repairable, so the vascular stumps were ligated. After extensive debridement, the talar extrusion was reduced by traction and countertraction maneuvers. Radiological imaging confirmed an adequate reduction ( Fig. 3 ). Definitive repair of the medial malleolar fracture and deltoid ligament was postponed for 48 hours, a period in which the patient continued a course of intravenous antibiotics and edema control. The final fixation of the tibial malleolar fracture was performed using 2.7-mm cannulated screws with washer. Additional capsulorrhaphy and deltoid ligament repair of the proximal and distal stumps using Vicryl 1-0 (Johnson & Johnson, New Brunswick, NJ, United States) was required to ensure clinical and radiological stability ( Fig. 4 ). Stability was evaluated with anterior drawer and forced varus-valgus maneuvers and was considered adequate. The patient was discharged 72 hours after the final procedure. Weight-bearing restriction was held for four months; then, progressive rehabilitation began, and complete weight bearing was authorized at six months. One year after the injury, the patient walks with no pain nor external aids, and X-rays reveal no signs of avascular necrosis ( Fig. 5 ).

## Discussion

To date, there is no consensus on the treatment of this injury, considering its relatively low presentation and the diverse results using different techniques and different follow-up periods. Regardless of the selected technique, the main goal of the treatment is to avoid infection, talar AVN, and posttraumatic arthrosis (PTA). 1 The treatment options consist of closed and open reduction, with or without reimplantation. Depending on the associated injuries, additional fixation may be required. Early tibiocalcaneal arthrodesis with excision of the talus has also been described. 11 12 Nevertheless, in case of open extrusion with no major signs of infection or severe contamination, early reimplantation can be attempted, and it must be the first treatment option. 6

In closed talar dislocations with no soft-tissue injury, closed reduction might be attempted followed by cast immobilization for four to eight weeks. 8 9 If there is interposed tissue that makes the reduction impossible, open reduction should be attempted. Hindfoot stability should always be tested after reduction (and after the internal fixation of associated fractures). In cases of instability, either external fixation or percutaneous pinning may increase support. 6 According to Weston et al., 6 results are adequate with both closed and open reduction, even if AVN develops. In the case herein reported, the results were evaluated by X-ray findings and by the patient's report of his condition. No functional scale was used, as there is no adequate score validated in Spanish for this specific condition.

The pseudoarthrosis technique has been presented as an option to avoid early arthrodesis, reducing infection rates, and aiming for an early recovery. 11 12 Though infection rates are low in arthrodesis and pseudoarthrosis procedures, the functional results are affected by the pitfall of producing a limb length discrepancy of up to 4 cm in the affected ankle, which may compromise the gait cycle. 10 11 12 For these reasons, it is reserved for the treatment of difficult cases and secondary complications.

Reduction of the extruded talus should always be attempted as the first treatment option, as it reduces the risk of AVN and PTA. Even in open extrusions, early or delayed reimplantation is associated with adequate functional results. Arthrodesis compromises ankle functionality; therefore, it should be a last resource.
