# Supplementary material for: Early Reduction of an Open Extruded Talus: Case Report
Source: Rev Bras Ortop (Sao Paulo). 2022 Apr 25;59(Suppl 2):e233–7. doi: 10.1055/s-0042-1744489 (PMC11679702; doi:10.1055/s-0042-1744489)
Supplement: Related texts

# Research article: Redução precoce de tálus em extrusão aberta: Relato de caso

**Keywords:** fraturas ósseas, infecções, necrose avascular, tálux

## Abstract

A luxação do tálus é uma lesão infrequente e com desfechos variáveis em relatos e séries de casos. Sua epidemiologia ainda não foi esclarecida, pois a lesão é descrita de diferentes formas: extrusão completa do tálus, luxação fechada ou aberta, luxação aberta com fratura do tálus, ou luxação aberta com fratura de maléolo. Tais classificações limitam a possibilidade de avaliação desta lesão como uma patologia única. Também não há consenso sobre o melhor tratamento para a luxação do tálus. Diversas técnicas de tratamento foram descritas, inclusive reimplante com e sem fixação externa, osteossíntese precoce, e até mesmo talectomia e pseudoartrodese tibiocalcânea precoces. Os desfechos desse tipo de lesão podem variar tanto quanto as opções terapêuticas. Entre as complicações no primeiro ano após a lesão, estão necrose avascular (NAV) e osteoartrite pós-traumática precoce. Este trabalho relata o desfecho funcional e radiológico adequado um ano após a redução precoce de uma extrusão completa do tálus com osteossíntese de uma fratura do maléolo medial.

## Introdução

As extrusões de tálus são causadas por traumatismos de alta energia, e são pouco relatadas na literatura. Estima-se que respondam por 0,06% de todas as luxações e 2% de todas as lesões talares. 1 2 3 A extrusão do tálus foi descrita pela primeira vez em 1680 por Fabricius Hildanus; séculos mais tarde, em 1919, Anderson a chamou de “tálus do aviador” depois de observar esse padrão de lesão em pilotos que sofreram acidentes de avião. 4 Embora muitos padrões de lesão sejam classificados como “luxação do tálus”, a extrusão do tálus é definida como a dissociação completa desse osso das articulações tibiotalar, talonavicular e talocalcânea. De modo geral, é acompanhada por fraturas do tálus ou do maléolo, e, às vezes, por ferida. 5 6

As características anatômicas únicas do tálus podem predispor a certas lesões e complicações, como necrose avascular (NAV). 6 A presença de múltiplas superfícies articulares (60% a 80% do osso são cobertos por cartilagem) limita a superfície óssea para vasos nutrícios, e a ausência de inserções musculares torna o tálus mais suscetível a luxação em traumatismo de alta energia. 7 8 9

Embora até o momento não haja consenso sobre o tratamento ideal da extrusão isolada do tálus, reimplantes precoce e tardio com ou sem fixação complementar têm sido relatados. 1 2 3 7 8 10 11 12 Por isso, apresentamos o caso de um homem adulto com extrusão completa do tálus após traumatismo de alta energia, que foi tratado com reimplante agudo e fixação da fratura do maléolo medial.

## Relato de Caso

Este trabalho foi aprovado pelo Comitê de Ética em Pesquisa do Hospital Universitario de la Samaritana.

Um homem de 26 anos apresentou uma extrusão aberta do tálus após um acidente de moto. Havia uma ferida de 10 cm na face medial do tornozelo, pela qual o tálus sofreu uma extrusão parcial ( Fig. 1 ). O paciente estava hemodinamicamente estável, sem outras lesões à primeira avaliação. O pé apresentava sensibilidade adequada e perfusão distal por pulso podal palpável, mas não por pulso tibial posterior. As primeiras radiografias revelaram uma fratura maleolar e tibial transversa e luxação medial do tálus a 270°; a tomografia computadorizada não revelou outras fraturas no tálus ( Fig. 2 ). O tratamento inicial consistiu em antibióticos (cefazolina, gentamicina, e penicilina G) administrados por via intravenosa, além de desbridamento e redução aberta em centro cirúrgico oito horas após o acidente. A exploração cirúrgica revelou secção completa do ligamento deltoide, da artéria tibial posterior, e da veia tibial posterior. Como esta lesão vascular não era passível de reparo, os cotos vasculares foram submetidos a ligadura. Após desbridamento extenso, a extrusão do tálus foi reduzida por manobras de tração e contratração. A imagem radiológica confirmou a redução adequada ( Fig. 3 ). A correção definitiva da fratura do maléolo medial e do ligamento deltoide foi adiada por 48 horas; nesse período, a antibioticoterapia intravenosa e o controle do edema foram mantidos. A fixação final da fratura maleolar tibial foi feita com parafusos canulados de 2,7 mm com arruela. Uma capsulorrafia adicional e o reparo dos cotos proximal e distal do ligamento deltoide com Vicryl 1-0 (Johnson & Johnson, New Brunswick, NJ, Estados Unidos) foram necessários para garantir estabilidade clínica e radiológica ( Fig. 4 ). A estabilidade foi avaliada com as manobras de gaveta anterior e varo-valgo forçado, e considerada adequada. O paciente recebeu alta 72 horas após o último procedimento. A restrição de carga foi mantida por quatro meses; a reabilitação progressiva foi iniciada em seguida, e a descarga completa de peso foi permitida aos seis meses. Um ano após a lesão, o paciente caminha sem dor nem auxílios externos, e as radiografias não revelam sinais de NAV ( Fig. 5 ).

**Fig. 1:** ( **A** , **B** ) Extrusão aberta completa do tálus em 270° de rotação, sem evidência de fratura. ( **C** ) Os vasos tibiais posteriores apresentam trombose e não são passíveis de reparo (seta).

**Fig. 2:** A radiografia mostra a luxação completa do tálus e a fratura maleolar e tibial em incidências anteroposterior, de mortise, e de perfil ( **D-F** ). A tomografia computadorizada detalha a ausência de fraturas no tálus e revela a luxação medial completa em projeções coronal, axial e sagital ( **G-I** ).

**Fig. 3:** Radiografia pós-redução. O tálus recuperou sua posição, e a fratura maleolar e tibial é agora mais evidente (setas).

**Fig. 4:** Radiografia após redução e fixação final da fratura maleolar e tibial 48 horas após o primeiro procedimento.

**Fig. 5:** Radiografia com sustentação de peso em um ano de acompanhamento. Não há evidência de necrose avascular da cúpula do tálus, nem sinais precoces de artrose tibiotalar. A fratura maleolar e tibial apresenta consolidação completa.

## Discussão

Até o momento, não há consenso sobre o tratamento dessa lesão, considerando sua incidência relativamente baixa e os diversos desfechos de diferentes técnicas e períodos de acompanhamento. Independentemente da técnica escolhida, o principal objetivo do tratamento é evitar infecção, NAV do tálus e artrose pós-traumática (APT). 1 As opções de tratamento consistem em redução fechada e aberta, acompanhada ou não por reimplante. Maior fixação pode ser necessária dependendo das lesões associadas. A artrodese tibiocalcânea precoce com excisão do tálus também foi descrita. 11 12 No entanto, no caso de uma extrusão aberta sem sinais de infecção ou contaminação grave, o reimplante precoce pode ser tentado, e deve ser a primeira opção de tratamento. 6

Nas luxações fechadas do tálus sem lesão de tecidos moles, é possível tentar a redução fechada seguida de imobilização gessada por quatro a oito semanas. 8 9 Se o tecido interposto impossibilitar a redução, deve-se tentar uma redução aberta. A estabilidade do retropé sempre deve ser avaliada após a redução (e depois da fixação interna de fraturas associadas). Em casos de instabilidade, a fixação externa ou pinagem percutânea podem aumentar a sustentação. 6 De acordo com Weston et al., 6 os desfechos de reduções fechadas e abertas são adequados mesmo se houver desenvolvimento de NAV. Em nosso caso, os desfechos foram avaliados pelos achados radiográficos e pelo autorrelato do paciente sobre sua condição. Uma escala funcional não foi utilizada porque não há sistema adequado validado em espanhol para esta lesão específica.

A técnica de pseudoartrose é uma opção para evitar a artrodese precoce, pois reduz as taxas de infecção e visa a recuperação mais rápida. 11 12 Embora as taxas de infecção dos procedimentos de artrodese e pseudoartrose sejam baixas, os desfechos funcionais são influenciados pela discrepância de comprimento do membro de até 4 cm no tornozelo acometido, o que pode comprometer o ciclo da marcha. 10 11 12 Por isso, essa técnica é reservada para o tratamento de casos difíceis e complicações secundárias.

A redução do tálus em extrusão deve sempre ser tentada como primeira opção de tratamento, pois reduz o risco de NAV e APT. Mesmo em extrusões abertas, o reimplante precoce ou tardio está associado a desfechos funcionais adequados. A artrodese compromete a funcionalidade do tornozelo, e, por isso, deve ser considerada o último recurso.
